# Uptake and Elimination of Brevetoxin in Blood of Striped Mullet (*Mugil cephalus*) after Aqueous Exposure to *Karenia brevis*

**DOI:** 10.1289/ehp.7274

**Published:** 2004-09-23

**Authors:** Ricky T. Woofter, Kirsten Brendtro, John S. Ramsdell

**Affiliations:** Marine Biotoxins Program, Center for Coastal Environmental Health and Biomolecular Research, National Oceanic and Atmospheric Administration–National Ocean Service, Charleston, South Carolina, USA

**Keywords:** blood, brevetoxin, radioimmunoassay

## Abstract

There is a critical need to simply and reliably monitor brevetoxins routinely in the blood of humans and aquatic animals. We used striped mullet as laboratory test animals to better define the uptake and elimination kinetics of brevetoxin during an aqueous exposure to the brevetoxin-producing dinoflagellate *Karenia brevis*. Striped mullet were first exposed to sublethal densities of *K. brevis* (~ 250,000 cells/L) for 1, 4, 8, 12, and 24 hr. No mortality was observed in the aquaria, and at each time point blood samples were taken and applied to blood collection cards for brevetoxin analysis using radioimmunoassay (RIA). The RIA indicated that blood levels of brevetoxin (PbTx-3) increased to values significantly different from that of the controls at all five time points during exposure (*p* < 0.05). Striped mullet were then exposed to a *K. brevis* culture with a known brevetoxin concentration of 0.5 ng/mL. Even after exposures at a low brevetoxin concentration, RIA was able to detect 2.25 ± 0.62 ng/mL PbTx-3 equivalents in the blood of the mullet at 8 hr of exposure. When exposed to higher brevetoxin concentrations (3.5 and 5.4 ng/mL), blood brevetoxin increased to peak levels at 12 hr and then reached equilibrium after 24 hr in the continued presence of *K. brevis*. During this time of equilibrium, the mullet maintained brevetoxins with a blood:water coefficient of 2.2. To define the elimination of brevetoxin, striped mullet were next exposed for 8–10 hr and then transferred to fresh seawater containing no *K. brevis* for up to 116 hr. Blood brevetoxin levels remained elevated and decreased only by 50% 116 hr after transfer. The rate of elimination fit best to a two-phase exponential decay with a biologic half-life of 12 and 266 hr. This study, using RIA in conjunction with blood collection cards, demonstrates an effective means to monitor blood brevetoxin levels in finfish and provides a foundation to characterize biologically relevant levels of brevetoxin in other species impacted by red tide events.

Red tides have been documented on the Gulf Coast of Florida as early as 1530 (Taylor 1917). They occur nearly annually and often persist for many months (Woodcock 1948). The causative organism for these events, *Karenia brevis* (formerly *Gymnodinium breve* and *Ptychodiscus brevis*), produces a family of neurotoxins, collectively called brevetoxins (Davis 1948; Lin et al. 1981; Martin and Chatterjee 1969; Poli et al. 1986). Exposure to high densities of *K. brevis* (100,000–250,000 cells/L seawater) can cause fish kills (Quick and Henderson 1974; Steidinger and Joyce 1973). Brevetoxins from red tides are linked to deaths in marine mammals, including dolphins and manatees, which are intoxicated through both ingestion of organisms harboring high brevetoxin concentrations and inhalation of aerosolized brevetoxins (Landsberg and Steidinger 1998). Brevetoxins produced by *K. brevis* blooms also pose a risk to human health. Aerosol forms of the toxin are produced by wind and wave action and move onshore, causing transient respiratory irritation in people that inhale the toxin (Pierce 1986; Pierce et al. 1990). Humans can also experience the more severe symptoms of neurotoxic shellfish poisoning (NSP) as a result of consumption of molluscan shellfish that have accumulated brevetoxins (McFarren et al. 1965).

Blooms of *K. brevis* are regularly monitored to control health hazards associated with shellfish consumption. Bans on shellfish harvesting are initiated when *K. brevis* densities surpass 5,000 cells/L seawater (Landsberg and Steidinger 1998). Added significance lies in the fact that sustainability of shellfish aquaculture is at stake because of ecologic problems in harvesting areas. A better monitoring strategy will be a major factor in improving aquaculture practices and help control the hazards of toxin exposure. Biomonitoring, using readily collected biological fluids of target or sentinel species, permits the determination of biologically relevant toxin levels in living animals. Blood collection cards have provided a format for the simple collection, storage, and extraction of whole blood for detection of brevetoxins in laboratory mice that is compatible with biological (receptor assay) and instrumental (liquid chromatography–mass spectrometry) detection methods (Fairey et al. 2001). Recently, Woofter et al. (2003) developed a brevetoxin radioimmunoassay (RIA) that has improved the sensitivity of brevetoxin detection to < 2 ng/mL in whole blood. Because of the RIA’s higher sensitivity, doses 10 times less than those that elicit symptoms could be detected, and at higher levels of exposure, brevetoxins could be detected for at least 2 days. This RIA also had an added advantage for studies involving exposure to the predominant, less stable brevetoxin congener PbTx-2 in that it appears to also detect longer-lived metabolic products of the parent brevetoxin molecules.

Previous toxicokinetic studies for brevetoxin have used exposure by intravenous, intraperitoneal, intratracheal, and oral administration to laboratory mice and rats (Benson et al. 1999; Cattet and Geraci, 1993; Poli et al. 1990; Woofter et al. 2003). It was necessary to further these studies with marine species and with an exposure paradigm that incorporates contact with the causative organism, *K. brevis*. Exposing striped mullet (*Mugil cephalus*) to *K. brevis* in laboratory aquaria permits respiratory and oral exposure as well as dermal contact with the toxin-producing organism. Exposure to the toxin-producing species is important because *K. brevis* produces at least nine brevetoxin analogs, predominantly PbTx-2, a congener highly susceptible to metabolism (Plakas et al. 2002). Striped mullet is a widespread and abundant teleost species that inhabits estuaries and salt marshes as well as the open ocean (Collins 1985), where contact with *K. brevis* blooms is likely.

For this study, we exposed striped mullet to simulated blooms of *K. brevis* in laboratory aquaria. Brevetoxin accumulation in blood of the mullet over various lengths of exposure to *K. brevis* was used to determine the kinetics of uptake. Low-level exposures were also conducted to determine the lowest quantifiable levels of measurement. Finally, we performed a depuration study to determine the rate of brevetoxin elimination. The results demonstrate that mullet quickly accumulate brevetoxins in their blood and retain detectable brevetoxin levels many days after exposure to toxin has ended. This information provides a laboratory-based indication of the uptake of brevetoxin in fish that encounter a red tide, the biologically relevant levels that bathe tissues via the circulation, and an estimate of how long they disperse toxicity to upper trophic levels of the food chain after they leave the red tide. It is anticipated that this work will provide the opportunity to predict the extent of brevetoxin toxicity beyond the temporal and spatial bounds of an actual red tide event.

## Materials and Methods

### Striped mullet collection and maintenance.

Striped mullet (*Mugil cephalus*) between 10 and 20 cm in length were collected using both seine netting and cast netting in control estuarine creeks not known to experience *K. brevis* blooms, near Charleston Harbor, South Carolina. The mullet were transported to the laboratory in aerated coolers and held for 10 days to ensure viability. They were held in a 950-L specimen tank with constant filtration and aeration using a 20-L Eheim filtration system (Eheim GmbH & Co KG, Deizisau, Germany). The salinity of the sea-water was maintained at 20 ppt. The fish were fed Seaweed Selects Green Marine Algae (Ocean Nutrition, Salt Lake City, UT) daily.

### Algal cultures.

In exposure 1 we used *K. brevis* cells of the SP2 strain. The cells were grown in a batch culture using 10-L Bellco spinner flasks (Bellco Glass, Inc., Vineland, NJ) containing L-1–enriched sea-water (Guillard and Morton 2003). *K. brevis* cell densities in culture were counted with a Multisizer 3 Coulter Counter (Beckman Coulter, Miami, FL).

Exposures 2–5 were performed with the Wilson isolate of *K. brevis.* The cells were maintained in 1-L batch cultures enriched with f/2 medium (Guillard 1973) with the following modifications to the trace metals solution: ferric sequestrene was used in place of EDTA·Na_2_ and FeCl_3_·6H_2_O, and 0.01 μM selenous acid was added.

All cultures were maintained at 25 ± 1°C on a 16:8-hr light:dark cycle with autoclaved, 20-μm-filtered 36% seawater obtained from the seawater system at the Florida Institute of Technology field station (Vero Beach, FL). Cool white lights provided a photon flux density of 150–175 μE/m^2^/sec. The cultures were harvested for use in exposure experiments within the mid to late log phase of growth. RIA of the culture was performed to assess the total brevetoxin concentration in the culture and expressed in nanogram per milliliter PbTx-3 equivalents.

### Mullet exposure design.

A primary range-finding method (exposure 1), and later a cell toxicity method (exposures 2–4), was used for the 24-hr mullet exposure to *K. brevis*. In exposure 1, two glass rectangular 60-L treatment tanks were set up in a fume hood with three to four fish per tank. Fish were allowed to acclimate to the 20-ppt treatment tanks for 24 hr before exposure. Control fish were removed after this 24-hr period, and the *K. brevis* culture was added to the treatment tanks to an approximate density of 250,000 cells/L water. The fish were removed from each treatment tank after the desired exposure time (1, 4, 8, 12, and 24 hr).

Exposures 2–4 were conducted in four round 60-L treatment tanks with five fish per tank. One fish from each tank was removed before administration of *K. brevis* cells and served as a control. The culture was then divided evenly among the exposure tanks to expose fish to desired concentration of brevetoxin (0.49–5.54 ng/mL). One fish per tank was removed and bled at each time point (4, 8, 12, 24, 36, and 48 hr), at which time a 50-mL water sample was taken from each tank to determine the total, intracellular, and extra-cellular brevetoxin concentration.

To determine the elimination of brevetoxin, tanks for exposure 5 were set up and dosed (5.54 ng/mL PbTx-3 equivalents) as per exposures 2–4 except after 10 hr of exposure to the toxic culture, the fish were then transferred to tanks containing no *K. brevis*. At each time point (16, 26, 38, 72, and 116 hr posttransfer), fish were removed from the tanks and their blood sampled for toxin analysis.

### Blood collection.

At each time point, the mullet were anesthetized with 0.15 g/L MS-222 (3-aminobenzoic acid ethyl ester) until motionless. Blood was collected from the dorsal vein using a heparinized (lithium salt heparin, 70 mg/mL) 1-mL syringe with a 27-gauge needle.

Whole blood samples were applied to the grade 903 cellulose filter paper blood collection cards (Schleicher & Schuell, Keene, NH). Blood (100 μL) was applied to each circle on the blood-collection card (Adam et al. 2000). The cards were then allowed to dry overnight in a cool, dark environment. Once the cards were dry, they were separated by 6 in. × 6 in. weighing paper and transferred to airtight plastic bags (both from VWR Scientific Products, Suwanee, GA) containing desiccant packages and humidity cards (both from Multisorb Technologies Inc., Buffalo, NY). The blood collection cards were stored at −20°C until analyzed.

### Brevetoxin extraction from blood collection cards.

The dried blood spots were prepared and processed as previously described (Fairey et al. 2001). Briefly, the entire 100 μL dried blood spot was cut from the cellulose blood collection card and extracted overnight in 2 mL methanol with an extraction efficiency of 84 ± 2.4% for the PbTx-3 congener. Extraction efficiency and stability for brevetoxin metabolites on blood collection cards is unknown. The spots were removed, and the methanol extracts were brought to dryness with nitrogen using a Turbovap LV evaporator (Zymark, Hopkinton, MA) and then stored at −20°C until use. The blood spot extracts were resuspended in RIA assay buffer containing 10% methanol.

### Brevetoxin extraction from seawater.

Total brevetoxin was extracted from the *K. brevis* culture samples and seawater samples in a separation funnel with 1 × 10 mL then 2 × 2.5 mL methylene chloride. The methylene chloride fractions were combined and dried with vacuum centrifugation using an SC210A Speedvac plus (Thermo Savant, Woburn, MA), then reconstituted in 1 mL methanol.

### Radioimmunoassay.

RIAs were performed using a sheep antisera prepared against a PbTx-2–fetuin conjugate (Garthwaite et al. 2001; Woofter et al. 2003). RIAs were run in 12 × 75 borosilicate glass tubes in phosphate-buffered saline (PBS) containing 137 mM NaCl, 8 mM Na_2_HPO_4_, 1.5 mM KH_2_PO_4_, 2.7 mM KCl, and 0.01% Emulphor-EL 620 (all from Sigma Chemical Company, St. Louis, MO, except for Emulphor, from GAF, New York, NY). The assay tubes consisted of PbTx-3 standard or blood spot extract (50 μL), anti-PbTx antiserum (1:4,000), [^3^H]PbTx-3 (0.4 nM), in PBS (final assay volume of 500 μL). The seven PbTx-3 standards ranged from 0.01 to 1,000 ng/mL. The PbTx-3 standards and blood spot extracts were allowed to pre-incubate in buffer at room temperature with the anti–PbTx-3 antibody for 1 hr before the [^3^H]PbTx-3 tracer was added. The tubes were placed on a Titramax 100 shaker (Heidolph Instruments, Cinaminson, NJ) and incubated 1 hr. Sac-Cel (Alpco Diagnostics, Windham, NH) was then added to the assay tubes to allow for the separation of bound and unbound brevetoxin. The bound antibody was filtered onto 25 mm glass fiber filters, and each assay tube was rinsed with PBS (3 × 2 mL) using a 48-sample Semi-Auto Harvester (Brandel, Gaithersburg, MD). The filters were placed in 5.0 mL Scinti-verse (Fisher, Suwanee, GA), and the radioactivity was counted on a Tri-Carb 3100TR Liquid Scintilation Counter (Packard-PerkinElmer, Wellesley, MA).

### Data analysis.

All concentrations and half-maximal effective concentration (EC_50_) values were determined using Prism Graph Pad 4.0 (GraphPad Software, Inc. San Diego, CA). When appropriate, we used Prism to run an analysis of variance to determine significance.

## Results

**Figure 1:**
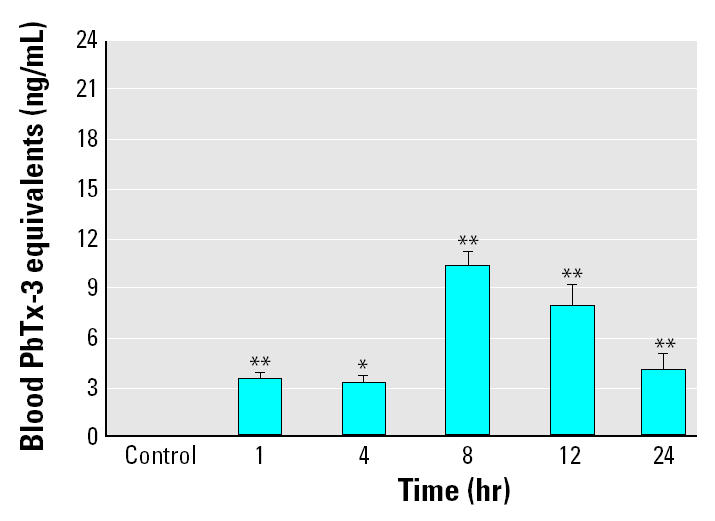
Blood brevetoxin levels after exposure to 250,000 *K. brevis* cells/L. Blood was collected from all four fish in each experimental group at each time point (0, 1, 4, 8, 12, and 24 hr). Blood brevetoxin levels reached a peak level of 10.37 ng/mL at 8 hr and then declined to 4.03 ng/mL after 24 hr exposure. The results shown are mean ± SE for four animals at each time point from a single experiment.
*p < 0.05 and **p < 0.01 compared with control.

The toxicokinetics of brevetoxin in finfish was determined by RIA of methanolic extract of dried blood stored on blood collection cards, a field collection method developed by Fairey et al. (2001) and adapted to RIA by Woofter et al. (2003). Because the blood kinetics of brevetoxin in finfish are not well characterized, we ran a preliminary exposure (exposure 1) in order to monitor the behavior of the fish and to optimize exposure time. During exposure 1, all fish at each time point were removed from the same aquarium, so these data reflect pseudoreplication. The blood brevetoxin levels in mullet exposed to 250,000 cells/L reached a peak level of 10.4 ± 0.84 ng/mL at 8 hr, and then declined to 4.03 ± 0.94 ng/mL after 24 hr exposure. We observed no behavioral changes and blood brevetoxin levels were significantly different from controls in all experimental groups (*p* < 0.01 at 1, 8, 12, and 24 hr; *p* < 0.05 at 4 hr; Figure 1). This exposure allowed us to evaluate the equilibrium of brevetoxin in the blood of striped mullet during a 24-hr exposure to 250,000 *K. brevis* cells/L.

Exposures 2–5 were run to estimate the limit of quantitation of blood brevetoxin in striped mullet, examine the relationship between internal and external dose, determine a long-term trend in blood brevetoxin levels, and estimate the toxicokinetics of brevetoxin in blood.

**Figure 2:**
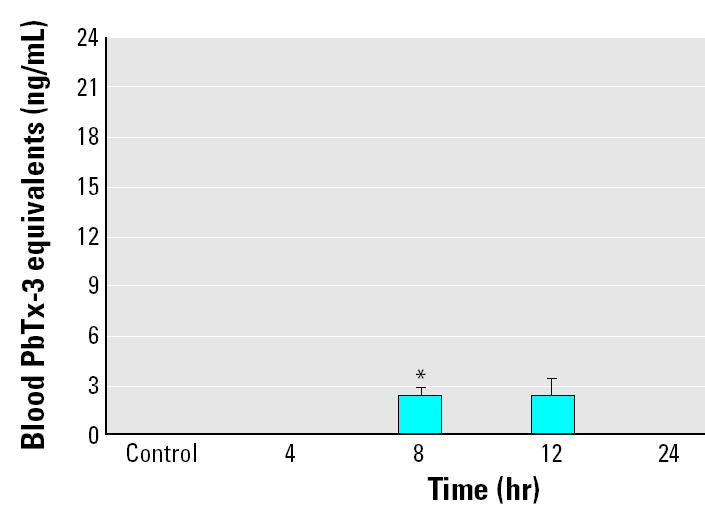
Blood brevetoxin levels after low-dose *K. brevis* exposure (0.49 ± 0.02 ng/mL). Blood was collected from one fish in each of the four exposure tanks at each time point (0, 4, 8, 12, and 24 hr). Detectable levels of brevetoxin were found in blood samples after 8 and 12 hr of exposure. The results shown are mean ± SE for four animals at each time point from a single experiment.
*p < 0.05 compared with control.

To estimate the limit of quantitation of blood brevetoxin in striped mullet after exposure to *K. brevis*, exposure 2 was run at a lower density of *K. brevis*, and the amount of toxin in the water was quantified at 0.49 ± 0.02 ng/mL PbTx-3 equivalents. Under these experimental conditions, detectable levels of brevetoxin were found in blood samples after 8 and 12 hr exposure but not at earlier (4 hr) or later (24 hr) times (Figure 2). After 8 hr exposure, the limit of quantifiable blood brevetoxin was 2.25 ± 0.62 ng/mL PbTx-3 equivalents (*p* < 0.05).

**Figure 3:**
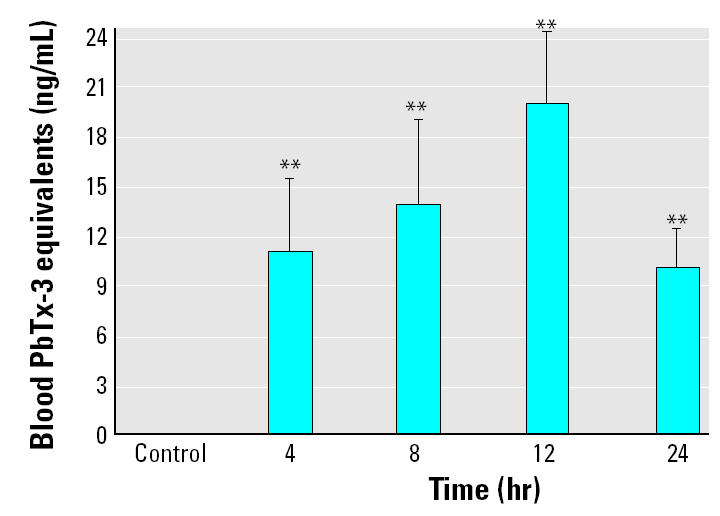
Blood brevetoxin levels after high-dose *K. brevis* exposure (3.49 ng/mL). Blood was collected from one fish in each of the four exposure tanks at each time point (0, 4, 8, 12, and 24 hr). Water toxicity remained unchanged (3.49 ± 0.20 ng/mL) throughout the course of the exposure, but blood brevetoxin levels increased to 19.23 ± 1.72 ng/mL at 12 hr and then declined to 9.63 ± 1.64 ng/mL after 24 hr exposure. The results shown are mean ± SE for four animals at each time point from a single experiment.
**p < 0.01 compared with control.

Next, we examined the relationship between internal and external dose of brevetoxin (exposure 3). For this exposure, we treated animals with a higher dose (3.49 ± 0.20 ng/mL) of brevetoxin containing *K. brevis* cell culture and measured both blood brevetoxin (internal dose) and tank water brevetoxin (external dose). At this higher dose, we observed a similar time dependency for blood brevetoxin levels as observed in exposures 1 and 2 (Figure 3). Blood brevetoxin levels were 19.23 ± 1.72 ng/mL PbTx-3 equivalents after 12 hr and then declined to 9.63 ± 1.64 ng/mL PbTx-3 equivalents after 24 hr exposure, with all blood levels being significantly different from controls (*p* < 0.01). However, the concentration of brevetoxin in the tank water remained constant for the duration of exposure and all other exposures.

**Figure 4:**
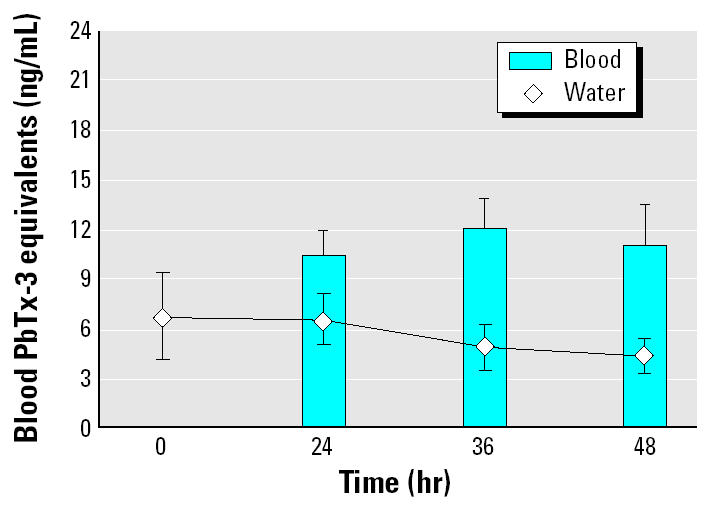
Blood brevetoxin levels after extended exposure to *K. brevis* (5.54 ± 0.58 ng/mL). Blood was collected from one fish in each of the four exposure tanks at each time point (0, 24, 36, and 48 hr). Water toxicity remained constant for all time points, and blood brevetoxin levels remained unchanged (*p* > 0.05 for 24, 36, and 48 hr). The results shown are mean ± SE for four animals at each time point from a single experiment.

Exposure 4 examined the long-term trend in blood brevetoxin levels, conducting treatments for up to 48 hr. For this experiment, we exposed mullet to 1,102,000 ± 2,100 *K. brevis* cells/L at 5.54 ± 0.58 ng/mL PbTx-3 equivalents for 48 hr. During this study, three fish out of a total of nine died during the first 10 hr of exposure, consistent with findings of Pierce (1993). After continuing exposure for 48 hr, we found that blood brevetoxin levels remained constant, with no significant difference between 24, 36, and 48 hr (*p* > 0.05; Figure 4). Comparing these plateau levels of blood brevetoxin with the external dose, animals were found to maintain approximately twice (2.20 ± 0.31) the water level of toxin in their blood.

**Figure 5:**
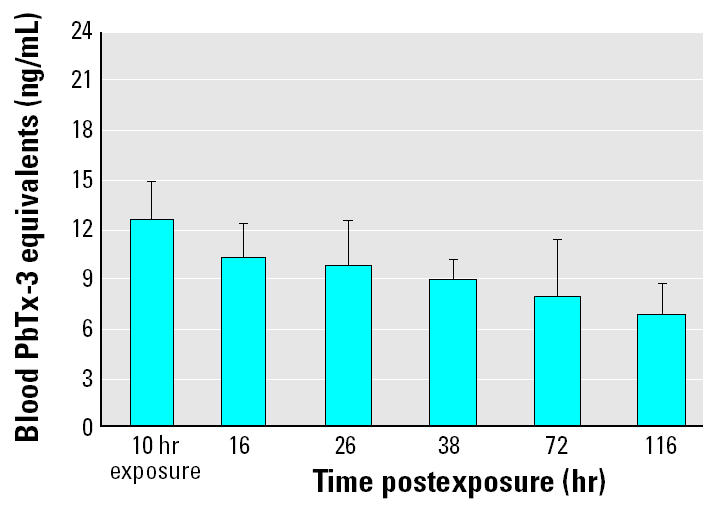
Elimination of blood brevetoxin after 10-hr exposure to *K. brevis* (5.54 ± 0.58 ng/mL). Fish were placed in control seawater after exposure. At each time point, 16, 26, 38, 72, and 116 hr postexposure, one fish per tank was removed for blood brevetoxin analysis. The results shown are mean ± SE for four animals at each time point from a single experiment.

As a final study, exposure 5 determined the elimination rate of brevetoxin from the blood of striped mullet. For this study, which was conducted in conjunction with the previously described extended exposure, mullet were removed from the *K. brevis*-treated tanks at 10 hr and placed in tanks containing control seawater. We chose to remove the fish at 10 hr because of the characteristic peak in blood brevetoxin levels between 8 and 12 hr of exposure. One fish was removed from each of their respective tanks at 10 hr to determine the level of blood brevetoxin accumulation before being transferred to control tanks. After being transferred to control seawater, one fish was removed per tank to be analyzed for blood brevetoxin levels at 16, 26, 38, 72, and 116 hr posttransfer. Blood brevetoxin levels decreased from 12.51 ± 2.3 ng/mL at 10 hr of exposure to 6.75 ± 1.92 ng/mL after 116 hr in control seawater (Figure 5).

To determine whether the blood brevetoxin elimination over time in striped mullet follows an exponential decay model, we applied our blood brevetoxin values to both a one-phase and a two-phase exponential decay model (Table 1). Because brevetoxin remained in the blood after 116 hr, we set the constraints to plateau at zero in order to calculate an approximate biologic half-life (*t*_1/2_). Using Prism software, the one-phase exponential decay model gave a *t*_1/2_ of 126.7 hr and an *R*^2^ value of 0.9118. When the data were analyzed by a two-phase exponential decay model, it yielded a *t*_1/2_−1 of 12.9 hr and *t*_1/2_−2 of 229 hr with an improved fit of *R*^2^ = 0.9968. Finally, to determine a theoretical longest time of detection of blood brevetoxin in animals once the exposure has ended we analyzed the data with a constraint set at our 2.25 ng/mL limit of quantitation in blood. The one-phase exponential decay analysis indicated that a maximal time limit of quantitation was 300 hr or approximately 12.5 days and the two-phase decay prolonged detection for ≥50 hr to 14.6 days.

## Discussion

The studies presented here provide a first-time characterization of brevetoxin uptake and elimination in vertebrates after exposure to *K. brevis*. Blood was chosen as the sample for toxin analysis, first, because it is in equilibrium with different tissues and, second, because it provides a useful biomonitoring application when using blood collection cards (Fairey et al. 2001). The present study characterizes the uptake and elimination of brevetoxin after a laboratory-based exposure designed to reflect a natural exposure of an endemic fish to a brevetoxin-containing red tide.

### Exposure of aquatic species.

Brevetoxins are a threat to numerous aquatic wildlife species including fish, waterfowl, and marine mammals (Landsberg 2002; Steidinger and Joyce 1973). According to Roszell et al. (1990), *K. brevis* produces primarily PbTx-2 during log growth phase but produces PbTx-2, PbTx-1, and PbTx-3 in the approximate ratio of 20:4:1, respectively. Aquatic species are of particular relevance because *K. brevis* is a fragile dinoflagellete that readily breaks, releasing toxin directly into the water or upon contact with inert or living objects (Tester et al. 2000). Aquatic species are susceptible to toxin by multiple routes of entry including gills/respiratory, oral/gastrointestinal, and dermal pathways. Physiologically based toxicokinetic (PBTK) models have been developed for organic chemicals to evaluate each of these routes of entry using several species of fish (Nichols et al. 1991, 1996, 2004). The striped mullet used for this study may be susceptible to all three routes of entry: toxin released from broken cells may enter through capillary plexi of the gills; toxin associated with cells or cell fragments is filtered through fine gill rakers into the oral cavity; and the mullet have a cutaneous surface area to volume ratio sufficient to permit a significant dermal absorption (Lien and McKim 1993; McKim et al. 1996) In the present study we used an experimental design that includes all likely routes of exposure to striped mullet, which are common in regions endemic to dense *K. brevis* red tides.

### Accumulation of brevetoxin.

Mullet show a near immediate uptake of brevetoxin into the blood upon exposure to brevetoxin-containing *K. brevis* culture applied via the aquarium water. Brevetoxin is measurable in the blood as early as 1 hr of exposure and rises to a peak between 8 and 12 hr. Brevetoxin levels then fall by about 50% to reach a plateau level at 24 hr; this plateau level is maintained for at least an additional 24 hr in the continued presence of the toxin. PBTK modeling of respiratory uptake of organic chemicals shows a near immediate single-order accumulation of contaminant that reaches a steady-state level in blood as early as about 24 hr, depending on the partitioning coefficient of the test compound (Nichols et al. 1990, 1991). Although we could measure brevetoxin at the earliest time point (1 hr) and a steady-state level was found at 24 hr, the kinetics differed in that a peak value was found between 8 and 12 hr. A peak accumulation at 8–12 hr was observed with PBTK modeling of oral exposures in fish (Nichols et al. 2004). Hence, the kinetics of brevetoxin accumulation after aqueous exposure to *K. brevis* cells also likely includes intestinal adsorption of the toxin. This oral route of exposure is consistent with toxicity of brevetoxin producing red tides to planktivorous fish such as mullet. Mullet have narrowly spaced gill rakers that aid in the filtration of particles such as microalgae from water. Current evidence indicates that the gill rakers serve to sort and concentrate particles using a crossflow filtration mechanism that promotes the travel of the particles to the esophagus (Sanderson et al. 2001). In the exposure tanks used for this experiment, the *K. brevis* cells quickly break; however, brevetoxin likely associates with these particles and would be processed by the gill rakers to enter the digestive track. Striped mullet also ingest sediment for trituration and were observed foraging on the bottom of the tank.

### Elimination of brevetoxin.

The elimination of toxin was determined experimentally by transferring fish at the peak time of exposure to water containing no toxin. Brevetoxin was detectable in the blood several days after removal of the toxin, reflective of a slow elimination rate. Accordingly, a one-phase elimination model yielded a *t*_1/2_ of 126 hr. Application of a two-phase elimination model yielded an improved fit of *R*^2^ = 0.9968 (vs. 0.9118 for one-phase model) and *t*_1/2_ of 12.9 hr and 229 hr. Several, more traditional, brevetoxin toxicokinetic studies have been reported using [^3^H]PbTx-3 in rats and the toadfish. As may be expected, intravenous exposure leads to very rapid blood elimination kinetics (Kennedy et al. 1992; Poli et al. 1990). However, oral administration of brevetoxin leads to sustained blood levels of brevetoxin for many days (Cattet and Geraci 1993). This much longer retention of blood brevetoxin after oral exposure is consistent with the present study in which mullet were exposed to *K. brevis* in the aquarium water, and suggests that brevetoxin is reabsorbed by the intestines during digestion as well as after biliary secretion.

The present study differed from the more traditional toxicokinetic studies in that exposure was designed to be more representative of an environmental exposure. An elimination study using aqueous exposure of oysters has been reported by Plakas et al. (2002), who compared exposure of the animals to purified *K. brevis* cultures and purified PbTx-2 and PbTx-3. In shellfish tissue, PbTx-3 remains largely intact, whereas the unstable aldehyde PbTx-2 is rapidly converted to PbTx-3 and cysteine conjugates. PbTx-3 was not metabolized and was eliminated from the animals within 2 weeks, whereas PbTx-2 was rapidly metabolized and the cysteine-PbTx persisted for 8 weeks after exposure. Comparison of the oyster and mullet studies is only of qualitative value; the elimination times cannot be directly compared because the toxin analysis was conducted on the whole oyster with 2 weeks as the earliest time point. Additionally, RIA of brevetoxin metabolites may not be assured as quantitative without further characterization. Nonetheless, it is likely that the slow elimination of brevetoxins from mullet exposed to *K. brevis* cultures may also be a reflection of differential elimination rates for PbTx-2 metabolites. The RIA shows equivalent specificity for both PbTx-2 and PbTx-3 (Woofter et al. 2003); however, its cross-reactivity with metabolites is under investigation.

### Internal dose and distribution.

The blood brevetoxin levels increased as a function of dose for the three dose experiments. Maximal blood levels reached nearly 20 ng/mL at 12 hr after a 3 ng/mL aqueous exposure, which did not cause observable symptoms. The decline of blood brevetoxin levels to a plateau value between 24 and 48 hr permitted a near-equilibrium analysis of an *in vivo* blood:water partition coefficient. This value of 2.2 is similar to values reported for ethyl acetate and three times lower than reported for tetrachloroethane in rainbow trout (Fitzsimmons et al. 2001). Measurement of toxin in blood is of particular value because blood levels are a dynamic reflection of tissue levels. Uptake studies of organic compounds in fish have indicated that ratios of blood to well-perfused tissues in fish are relatively constant and reflect near-equilibrium conditions (Nichols et al. 1990). Indeed, Cattet and Geraci (1993) demonstrated that blood levels of brevetoxin parallel levels in heart, kidney, lung, fat, muscle, testes, brain, and skin > 192 hr after oral exposure of PbTx-3 to rats. Brevetoxin levels in stomach and intestines, which at 6 hr were much higher, declined to plasma levels between 24 and 48 hr. Only liver retained higher levels of brevetoxin than found in plasma after 96 hr. Distribution studies to determine the percentage of body burden have been conducted in the toadfish after both intravenous and oral radiolabeled PbTx-3 exposure (Kennedy et al. 1992; Washburn et al. 1994). These studies reported similar distributions for both routes of administration by percent body burden and found toxin largely in the muscle, liver, bile, stomach, and intestines. Based on our initial findings of brevetoxin uptake and elimination, further studies to determine partitioning coefficients between tissues and blood should permit the evaluation of brevetoxin partitioning in fish tissues after environmental exposure to *K. brevis* and other aquatic species.

### Implications for monitoring.

The retention of brevetoxins in finfish has substantial ecologic implications and potential practical significance. Mullet represent an important vector in the marine food web, being a common source of food for marine waterfowl, game fish, and marine mammals. Monitoring vectors in the food web, such as mullet, may provide a means to estimate the halo effect of a red tide beyond the boundaries demarcated by the *K. brevis* organism. This information has potential to extend modeling studies for the causative organism to models that may predict the spread of toxicity and its impact on wildlife and protected species, providing forecasting information to resource managers. Our results indicate that the RIA analysis of mullet using blood collection cards can detect brevetoxin up to 12.5 days after cessation of exposure. This study, being the first to explore the toxicokinetics of *K. brevis* in marine vertebrates, will provide a foundation to characterize biologically relevant levels of brevetoxin in other species impacted by red tide events.

## Figures and Tables

**Table 1:** One- and two-phase exponential decay analysis of blood brevetoxin retention in striped mullet.

Analysis	One phase	Two phase
Best-fit values		
Span 1 (hr)	11.62	9.569
*K*1	0.005469	0.003025
Span 2 (hr)	—	2.927
*K*2	—	0.05392
Plateau	0	0
*t*_1/2_−1 (hr)	126.7	229.1
*t*_1/2_−2 (hr)	—	12.86
SE		
Span 1 (hr)	0.4713	0.9007
*K*1	0.0009164	0.0009323
Span 2 (hr)	—	0.8983
*K*2	—	0.02295
Goodness of fit		
*R*^2^	0.9118	0.9968

—, not available; *K*, constant used in evaluating *t*_1/2_.
